# Agent-based modelling in synthetic biology

**DOI:** 10.1042/EBC20160037

**Published:** 2016-11-30

**Authors:** Thomas E. Gorochowski

**Affiliations:** BrisSynBio, University of Bristol, Life Sciences Building, Tyndall Avenue, Bristol BS8 1TQ, U.K.

**Keywords:** agent-based modelling, cell populations, collective behaviours, synthetic biology

## Abstract

Biological systems exhibit complex behaviours that emerge at many different levels of organization. These span the regulation of gene expression within single cells to the use of quorum sensing to co-ordinate the action of entire bacterial colonies. Synthetic biology aims to make the engineering of biology easier, offering an opportunity to control natural systems and develop new synthetic systems with useful prescribed behaviours. However, in many cases, it is not understood how individual cells should be programmed to ensure the emergence of a required collective behaviour. Agent-based modelling aims to tackle this problem, offering a framework in which to simulate such systems and explore cellular design rules. In this article, I review the use of agent-based models in synthetic biology, outline the available computational tools, and provide details on recently engineered biological systems that are amenable to this approach. I further highlight the challenges facing this methodology and some of the potential future directions.

## Introduction

Synthetic biology aims to apply engineering principles to biological systems to enable the more rational design of novel functionalities. This has resulted in the engineering of cells able to perform complex computations [1,2], act as biosensors of disease [3] and, building on the success of metabolic engineering, sustainably produce valuable drugs and chemicals [4]. In most cases, it is impractical to construct and test every possible design of a synthetic biological system. To address this issue, mathematical modelling and computational simulations form an essential part of the design process. They enable large-scale *in silico* investigations into the robustness of specific designs, help to identify key parameters, and can filter out designs that are likely to be non-functional [5]. This reduces the costly and time-consuming laboratory work required to develop a functional system.

**Figure 1:**
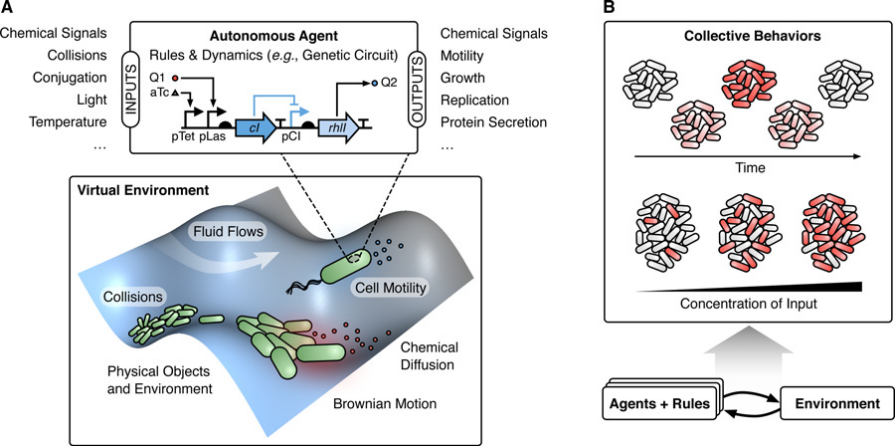
Principles of agent-based modelling (**A**) An agent-based simulation consists of a virtual environment where large numbers of autonomous agents can interact. A model of a bacterial colony is shown with agents representing cells. Each cell contains a synthetic genetic circuit that controls its behaviour. In this case, the genetic circuit takes two chemicals as inputs (Q1 and aTc) and produces a single chemical output (Q2) if both inputs are absent (a NOR logic operation). A range of common cellular inputs and outputs are shown. To ensure that simulations faithfully reproduce the biological system, key physical processes encountered or utilized by the agents must be implemented within the virtual environment. Those relevant to bacteria are shown. (**B**) Interactions between agents implementing specific rules and the shared environment can lead to the emergence of collective behaviours. These include dynamic co-ordination (e.g. synchronization of gene expression; see Figure 2A) and population-level encodings of continuous inputs (e.g. cells are either in an ‘ON’ or ‘OFF’ state and the fraction of the population in an ‘ON’ state corresponds to the continuous concentration of the input, similar to the bimodality of the lactose utilization network in *E. coli* [10]).

Owing to our ability to observe and measure many diverse aspects of individual cells, much of the modelling in synthetic biology to date has focused on intracellular dynamics (i.e. capturing changes in the rates of transcription and translation, and variations in the concentrations of chemicals, mRNAs and proteins over time). However, there is growing realization that the robustness of natural biological systems is often derived from collective population-level features that extend beyond individual cells. Colonies of bacteria are known to communicate and co-ordinate their growth during infection [6,7], and exploit collective behaviours to enable the emergence of antibiotic resistance [8]. To unravel these mechanisms and make use of them in our own synthetic systems, models must extend beyond intracellular dynamics and encompass the interactions between cells and their shared environment. Agent-based modelling (also referred to as individual-based modelling) attempts to bridge this gap by considering large numbers of autonomous ‘agents’ that can interact within a virtual environment [9] (Figure 1A). Agents can represent any entity of interest, such as a molecule, cell or multicellular organism, and each independently follows a prescribed set of rules. In a biological setting, these rules are often encoded as genetic circuits that drive cellular responses to particular stimuli. By simulating the behaviour of these virtual populations in realistic environments, it is possible to gain an understanding of how low-level cellular rules lead to the emergence of collective population-level behaviours [9] (Figure 1B).

A major benefit of using agents to model the discrete elements of a system is the ability to capture minor differences that exist or can arise between them. For example, intracellular noise causes the expression of the same protein to vary across a population, and for cells that are motile, differences in the history of their movement can lead to subtle changes in the way they respond to new stimuli. Other modelling approaches often average out these differences, assuming cells behave in a uniform way across the entire system. Although such simplifications are sometimes appropriate, many processes in biology actively make use of cellular differences to achieve novel functions. One of the most famous is the bimodality of the lactose utilization network in *Escherichia coli*. In this system, mixed populations can emerge with a cell being either fully active or inactive, with the fraction of both controlled by the lactose concentration [10] (Figure 1B). This is useful because a diverse population ensures that at least some cells are poised to exploit potential changes in environmental conditions, improving the fitness of the population as a whole. Averaging the state of cells across the population would miss this vital feature.

Another aspect of agent-based modelling that is difficult to reproduce using other methodologies is the multiple ways that interactions between agents can occur. The most basic type of interaction is physical, where two agents meet. However, because not all interactions may lead to a behavioural response, the rules controlling how an agent reacts are often probabilistic. This is akin to the chance that you might fall ill after meeting a colleague that is feeling unwell. In addition to direct encounters, the environment itself can also act as a means for indirect interactions [11]. In Nature, pheromones are often deposited into the environment to be sensed later by other individuals. This allows the environment itself to become an indirect channel for communication. In both cases, modelling the range of interactions that take place is a challenge for many methods, but is easily handled by agent-based models because these events are explicitly captured.

In this review, I discuss the general principles of agent-based modelling and show how it can support the rational engineering of collective behaviours in synthetic biology. Although agent-based modelling has begun to see applications in diverse areas of this field (e.g. in the design of co-operating nanoparticles for medicine [12]), in this review, I focus exclusively on cellular systems with agents representing individual cells. Recent synthetic biology examples are used to illustrate how population-level features can arise from simple cellular programs, and a full list of currently available computational tools will be provided. This review aims to give a general introduction to the field of agent-based modelling, some of its applications to synthetic biology and outline the challenges and future directions of this methodology.

## A brief history of agent-based modelling

The study of autonomous agents interacting within a virtual environment dates back to the start of computer science and the self-replicating machines proposed by von Neumann in the 1940s [13]. These were designed to mimic the process of replication that is fundamental to life. The machines took input materials and signals from their environment, and, through a predefined set of rules, created an identical copy of themselves.

The first use of the term ‘agent’ with the same meaning as in this review arrived much later, around the 1980–1990s [14]. During this period, increases in computing power made it feasible to simulate systems of a useful size, and rapid growth was seen in the number of tools available to support researchers in this area. Some of the most popular were based around the Logo programming language [15] (e.g. StarLogo [16] and NetLogo [17]) that was originally developed for use in teaching. Because of its simplicity, Logo was perfectly suited to allow anyone to define the rules of an agent-based simulation and study the emergent behaviours that could arise. Since then, the use of agent-based modelling has continued to expand, with the approach now extensively used in the fields of economics [18], social behaviour [19], ecology [20], microbiology [9] and epidemiology [21], as well as many others.

## Agent-based modelling in synthetic biology

Synthetic biology attempts to apply engineering principles to biological design. A core part of this process is the use of predictive mathematical models to test and optimize potential designs. In more established engineering disciplines, such as mechanical engineering, laws have been derived and differential equation models can be used to accurately predict the dynamics of a system. Although similar approaches translate to large biological systems (e.g. modelling the biophysics of animal movement), at the cellular level these methods often break down due to the discrete numbers of cells and molecules involved, and the inherent heterogeneity that arises from cellular noise. This places limitations on our ability to predictably engineer the precise behaviour of individual cells. Nature tackles this issue by using collective behaviours that are able to accommodate environmental and cellular noise. This allows for unreliable behaviours at the level of individual cells to be transformed into accurate and robust functions at a population-level. Examples include the use of quorum sensing to co-ordinate a response [22] and the synchronization of rhythmic processes [23]. Synthetic biology is beginning to develop systems that exploit collective behaviours, and agent-based modelling is ideally suited to describe these multi-scale systems and provide an effective framework for their study.

**Figure 2:**
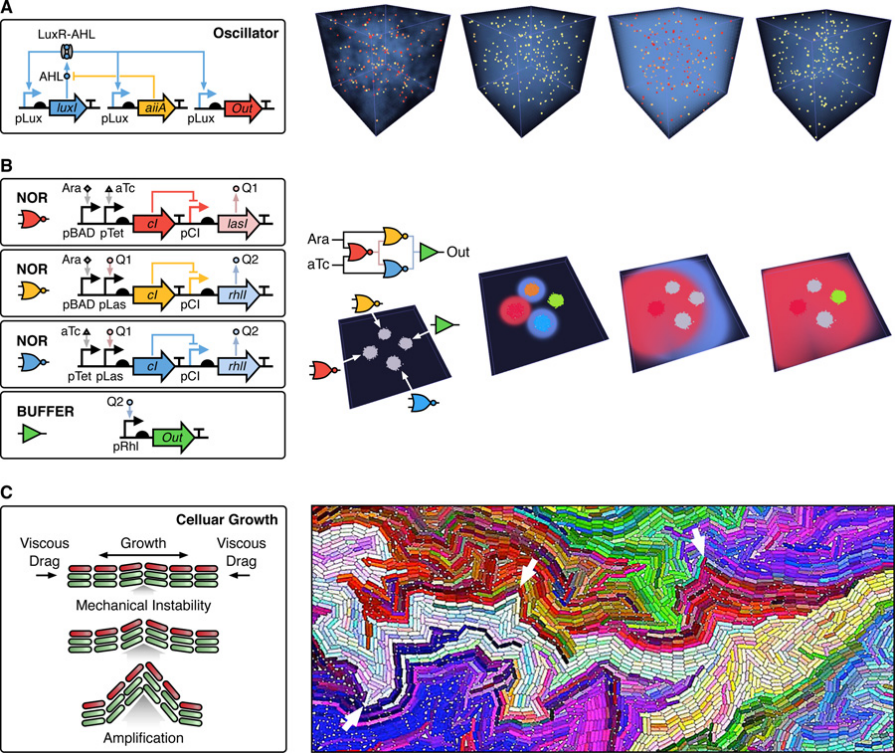
Agent-based models in synthetic biology. Boxes contain the physical rules or genetic circuit controlling the behaviour of each cell. Multicellular agent-based simulations are shown to the right illustrating the emergent behaviours that arise. (**A**) Robust synchronized oscillations across a population of cells [27]. Each cell encodes an identical genetic circuit able to generate oscillations in the expression of an output gene (*Out*). The *luxI* gene encodes an enzyme that catalyses the production of *N*-acylhomoserine lactone (AHL). AHL binds to the constitutively produced LuxR protein (not shown), which activates the pLux promoters. AHL can also diffuse through the cell membrane into the environment to affect other cells (shown by the blue semi-transparent fog), and is negatively regulated by the *aiiA* gene whose product degrades AHL. The simulation shows 200 cells (small spheres) that start with random initial expression levels of circuit genes in a 100 μm^3^ box with wrapping boundary conditions. The colour of each cell corresponds to the expression of the output (yellow=low; red=high) (**B**) Four spatially separated colonies that collectively implement an EQUAL logic function (output is active when both inputs are simultaneously inactive or active) by using diffusing quorum molecules as chemical wires for communication [36]. The genetic circuit for each colony is shown that implements either a NOR or BUFFER logic function. Arabinose (Ara) and anhydrotetracycline (aTc) are used as inputs to the circuit. Q1 and Q2 are the quorum-sensing molecules 3OC12-HSL and C4-HSL respectively. These are able to diffuse through the cell membrane into the environment and are shown by the red (Q1) and blue (Q2) semi-transparent fogs that propagate in the simulations. Each colony in the simulation consists of 20000 cells, which are coloured if the output promoter (pCI) is active. The simulation starts with all cells inactive and both arabinose and aTc absent from the medium. (**C**) Generation of fractal colony structures through accurate agent-based modelling of rod-shaped bacteria colony growth. The simulation image is adapted from http:://cellmodeller.org and shows cells coloured according to their mother–daughter relationship. On division, the daughter cell colour is chosen on the basis of its mother colour with a small random change. Several points of mechanical instability are highlighted with white arrows. Simulations in (**A**) and (**B**) were generated using BSim [39] and (**C**) using CellDesigner [63]. Genetic circuits are shown using Synthetic Biology Open Language Visual (SBOLv) notation [84] and generated using DNAplotlib [85].

Oscillations are a fundamental dynamic behaviour exhibited by many biological systems [24]. This has led to extensive interest in synthetic biology to understand the design rules of synthetic genetic circuits able to robustly oscillate [25]. One of the first successful examples was the ‘repressilator’ circuit that used a ring of repressor proteins to generate waves of activation [26]. Although this system was functional, the oscillations within single cells were fragile and displayed variable dynamics with differing amplitudes and periods. To address this issue, Danino et al*.* [27] showed how a simpler gene circuit that used a quorum-sensing molecule for regulation could produce sustained oscillations across entire populations of cells (Figure 2A). Critically, variability between cells was buffered by cell-to-cell couplings due to a shared quorum-sensing molecule that was able to freely diffuse across the population. It was shown that an improved cancer therapy could be produced by combining this oscillating circuit with the production of an anti-cancer drug and cell lysis system [28]. When this circuit was placed in a strain of bacteria that preferentially associated with cancerous tissue, it was found that the cells could act as an effective drug delivery vessel. Once a population had become established at a tumour site, waves of drug release were generated that significantly improved the efficacy of standard treatments such as chemotherapy [28]. An interesting property of this population-level oscillator was that small numbers of isolated cells did not display oscillations. Only when a sufficient number (a quorum) was reached did oscillatory dynamics emerge. To better understand this behaviour, Mina et al*.* [29] used agent-based modelling to assess the necessary conditions. They showed that oscillations could only arise in the presence of high concentrations of the quorum-sensing molecule, due to a difference in the timescales of key regulatory components within the oscillating circuit. Furthermore, the cyclic behaviour of large numbers of cells resulted in stronger coupling between individuals, which influenced the behaviour of the regulatory network within each cell. This highlighted the importance of considering potential environmentally mediated collective effects (e.g. chemicals that can diffuse between cells to couple their behaviours) when designing synthetic genetic circuits.

A significant difficulty when developing large synthetic genetic circuits is the burden that they place on the host cell [30]. Expression of large numbers of foreign genes can lead to growth defects and affect the normal behaviour of a cell [30–34]. To alleviate these issues, attempts have been made to separate parts of a circuit and place each sub-circuit into a different cell [35–38]. This division of labour reduces the burden placed on each individual cell and allows for optimization of these simpler parts. This approach was used by Tamsir et al*.* [36] to implement complex logic circuits that used ‘chemical wires’ to communicate the result from one part to the next (Figure 2B). Cells were engineered to implement several basic logic functions that used quorum-sensing molecules as inputs and outputs. More complex functions could then be generated by creating a circuit that consisted of spotted colonies of cells containing the required logic gates. Not only did this permit fewer synthetic genetic components per cell, but also it enabled the rapid reconfiguration of the circuit by merely altering the types of cell spotted and their positions. Because this system relies on the intracellular dynamics of each cell to affect the shared environment of the others (e.g. the production of a quorum-sensing molecule that freely diffuses between the cells), agent-based modelling is ideally suited to studying its behaviour. Agent-based simulations have been used to assess the limits of this approach and shown that, whereas generally the system functions robustly, the need for chemical diffusion leads to large propagation delays and requires that colonies are located closely in space to ensure that signalling molecules reach sufficiently high concentrations [39]. These limitations make such a method unsuitable for systems requiring a short response time. To alleviate this problem, engineered bacteria have also been designed using agent-based models to perform similar functions exploiting conjugation as a quicker means of direct cell-to-cell communication [40].

A major advantage of using agent-based simulations is their ability to accurately capture the physical interactions that occur between large numbers of cells. Because many types of cell have a non-isotropic shape (e.g. are rod-shaped), their normal growth and replication can lead to the emergence of intricate structures at the level of the colony. Agent-based models have been used to explore this feature as an area for future morphogenetic engineering applications [41,42]. Rudge et al*.* [42] implemented large-scale simulations of bacterial colonies to show that local mechanical instabilities will arise due to the uniaxial growth of cells and viscous drag from the environment (Figure 2C). Subsequent growth and division of cells at these points amplifies these instabilities and leads to ‘kinks’ that propagate through the colony to generate a fractal internal structure. This demonstrated the ability for physical interactions alone to lead to the emergence of complex population-level features. In addition to the study of colony substructure during normal growth, agent-based models have also been used to explore the self-organization of cells at high-cell densities [43] and to develop rules controlling growth that guide the emergence of desired colony morphologies [41].

Heterogeneity within an environment can also strongly influence the behaviour of a system. In bioreactors, it is often assumed that rapid stirring ensures a good mixing of cells and media. This greatly simplifies the modelling of reactors as they can be treated as a uniform entity where the availability of substrates is similar throughout. However, for large vessels, this assumption often breaks down due to the formation of eddies and other flow-related features that hinder proper mixing. Agent-based models have been used to investigate what effects these might have on the behaviour of cellular populations. Simulations have been used to follow the paths of individual cells within the turbulent flows and monitor the local fluctuations they experience [44]. Similar types of model have also been applied to algal systems to understand how aspects of a photo-bioreactor and the non-uniform growth of cells within it, influence photopenetration [45,46]. For these industrially focused systems, agent-based models provide vital information to help improve reactor design and optimize growth conditions to ensure maximal yields of a product are achieved.

## Agent-based modelling tools

There are numerous agent-based modelling tools and many provide features of direct relevance to synthetic biology (Table 1). Three of the most widely used agent-based frameworks are NetLogo [47,48], Repast [49,50] and the Flexible Large-scale Agent Modelling Environment (FLAME) [51]. These are all general-propose frameworks that provide minimal built-in functionality. Instead, they allow for extensive customization of agent behaviours and the environment itself. Repast and FLAME are also designed to produce highly scalable simulations that can be automatically optimized to run on systems ranging from desktop computers to high-performance computer clusters. Although such frameworks can be used to simulate a broad range of systems, their lack of biologically relevant built-in features means that significant effort is required to produce a working simulation. The requirement on a user to implement complex cellular traits (e.g. growth, replication and movement) as well as the environmental physics necessary to capture movement and interactions of cells means that such frameworks are generally only suitable for highly specific problems where customized implementations of many processes are necessary.

**Table 1.** Comparison of agent-based modelling tools

	Agent dynamics and features*										Environment					
Name	Simple rules	Advanced rules	ODEs	DDEs	Chemical equations	Stochastic dynamics	Motility	Chemotaxis	Cell replication	Cell morphology	2D	3D	Chemical diffusion	Complex objects†	Language	Reference(s)
AgentCell					●	●	●	●				●			Java	[53]
BacSim‡		●			●	●			●		●		●		Obj-C/Java	[55,56]
BNSim	●	●			●	●	●	●	●			●	●		C++	[62]
BSim	●	●	●	●		●	●	●	●			●	●	●	Java	[39]
CellModeller	●	●	●			●			●	●		●	●		Python§	[63]
Chaste	●	●	●	●	●	●			●	●	●	●	●	●	C++	[65,66]
CompuCell3D	●	●	●			●	●	●	●	●	●	●	●	●	C++/Python	[67]
DiSCUS	●	●	●						●	●	●				C	[51]
FLAME	●	●				●			●		●				Python	[40]
gro	●	●				●	●	●	●	●	●		●		C++	[64]
iDynoMiCS		●			●	●			●		●	●	●		C++	[58]
NetLogo	●						●		●		●	●			Scala║/Java	[47,48]
Organism	●	●	●						●	●		●	●		C++	[43]
RapidCell					●	●	●	●			●				Java	[54]
Repast HPC	●	●									●	●			C++	[50]
Repast Simphony	●	●									●	●			Java	[49]

* Columns are defined as follows. Simple rules, a limited subset of commands are available to control agent behaviours; Advanced rules, access to a full programming language is provided to control agents; ODEs, agents can use ordinary differential equations to describe their internal state; DDEs, agents can use delay differential equations to describe their internal state; Chemical equations, cellular chemical reaction networks can be simulated; Stochastic dynamics, the internal state of an agent and the interactions with other agents can be stochastic, i.e. upon meeting another agent, there is a probability that they interact; Motility, agents can move freely within the environment and functionality to manage collisions/interactions is available; Chemotaxis, a realistic implementation of chemotaxis is available to control cellular movement; Cell replication, agents are able to replicate over time; Cell morphology, agents can take an arbitrary shape or have the option to take one of multiple predefined shapes.

† Ability to define solid structures within the environment that have arbitrary geometries.

‡ BacSim is no longer developed and has been superseded by iDynoMiCS.

§ Simulations are accelerated using the PyOpenCL library, which provides access to parallel computation on GPUs.

║ Scala code is compiled into Java byte-code to enable full interoperability with Java tools and other JVM-based languages.

Cellular motion is often a major determinant of the physical interactions that take place across a population, and chemotaxis is used by many types of cells to move and navigate chemical concentration gradients within their environment [52]. Several agent-based tools have been developed to simulate and analyse this behaviour. The AgentCell [53] software implements the entire chemotaxis biochemical network of *E. coli* and provides a physically realistic three-dimensional environment for cellular movement. The tool is built using Repast [49] and includes a fully stochastic simulator for the biochemical reaction networks within each cell. By coupling detailed cell models to environmental properties, such as chemoattractant gradients, the resulting agent-based models can reproduce many experimentally observed features at both the level of single cells and the population. Inspired by AgentCell, the RapidCell [54] software also simulates a population of motile *E. coli* cells, but within a simplified two-dimensional environment. Rather than performing a fully stochastic simulation of the entire chemotaxis biochemical network, it employs a hybrid simulation approach. This mixes algebraic and differential equations to model the fast and slow reactions respectively, whereas major events such as flagellar motor switching are handled using stochastic methods. This significantly reduces the computational demands, allowing for up to 1 million cells to be simulated on a standard desktop computer, with results that still accurately match experimental observations.

A general area of biology that has seen extensive use of agent-based modelling has been the study of biofilm formation. The BacSim [55,56] software was one of the first agent-based tools to study biofilm growth and asses the role of heterogeneity within these populations. Biologically verified rules relating to substrate uptake, metabolism, maintenance and growth are implemented within each cell, and simulations take place in an environment that allows for the diffusion of substrates. The growth observed in BacSim closely fitted another widely used biomass-based model [57]. Following this work, a new tool called iDynoMiCS [58] was developed to supersede BacSim, implementing a more detailed three-dimensional environment and including many improvements such as pressure fields to enable the contraction or spreading of biofilms over time, and more realistic fluid behaviour of the extracellular matrix. This model has since been used to test the effect of physical and biological factors on biofilm growth and the role of quorum-sensing inhibition as a way to disrupt their structures [58–61].

In addition to these highly specific tools, a number of more general agent-based frameworks are also available that include biologically relevant elements to ease the development of new models. The Organism [43] software is one of the simplest, allowing for standard ordinary differential equation (ODE) models of general biochemical reaction networks and mechanical rules within and between cells. The BSim [39] software provides a broad range of features. These include a physically realistic three-dimensional environment that implements Brownian motion, diffusive chemical fields, and the ability to include multiple forms of agent within a single simulation. Agent dynamics can take many forms with simulators provided for ODEs, delay differential equations (DDEs) and general rule-based dynamics. BSim also provides a broad range of example simulations that can be adapted and combined to tackle a wide range of agent-based modelling tasks. The BNSim [62] software provides similar functionality, but also includes stochastic simulators that implement Gillespie's Exact SSA method and solvers for stochastic differential equations (SDEs). BNSim is also optimized to accelerate simulations through the efficient use of multi-core processors.

To capture the often rod-like shape of commonly engineered bacteria such as *E. coli*, several tools contain accurate cell shapes and models of growth and replication. CellModeller [63] software is designed to study the formation of synthetic biofilms and makes use of OpenCL (a high-performance computing library) to enable the efficient simulation of colonies containing more than 30000 cells. This is possible through the implementation of novel parallel algorithms that can rapidly compute the collisions and forces between cells. For agent dynamics, CellModeller provides simulators for both rule-based programs and ODE equations. The gro [64] software also realistically models bacteria as growing rods and makes use of its own high-level specification language called ‘gro’ to define simulation parameters and agent rules. This language is designed to simplify the expression of high-level rules, while still being capable of implementing any chemical reaction network or gene regulatory model. The gro language has already been used to describe a number of cellular rules that lead to the growth of diverse colony architectures and enable cells to sense their position within a colony [41].

For dense tissue-like environments, more specialized approaches are required to capture the complex geometries between cells as they grow. The cell-based Chaste [65,66] software includes extensive functionality and is able to simulate cell populations using lattice-based, cell-centre or vertex-based models for cell position and connectivity in one-, two- or three-dimensional environments. Furthermore, to account for changes in cell–cell adhesion, which affects tissue structure, the laws governing forces between cells can be modified. Detailed cell-cycle models are embedded within each cell and these can be altered to govern growth and death processes. Complex boundary conditions can also be accommodated, allowing for simulation of realistic environments that contain other structural features. Simulators are included for a full range of deterministic and stochastic models that can supplement existing cellular models of behaviour. CompuCell3D [67] software is also suitable for tissue-based systems and uses a cellular Potts model (CPM) for cell growth. This approach allows for highly complex cell morphologies and has been successfully used to capture the growth of many different types of tissue. Similar to Chaste, CompuCell3D includes a broad range of functionality and offers the novel ability to use Systems Biology Markup Language (SBML) models to control cellular behaviours. It also includes highly optimized parallel implementations of simulators and an entire set of supporting applications to simplify the development of large models. The main disadvantage of both Chaste and CompuCell3D is that they are unable to model sparse cellular systems in which cells are separated by large distances.

An important factor for many experimentalists when choosing an agent-based modelling tool is the ease of use and accessibility to non-programmers. At present, the majority of agent-based tools require some basic level of programming experience in order to define a working model. Repast and CompuCell3D do offer graphical user interfaces for model creation, but these unfortunately lack any features of relevance for synthetic biologists. One of the major reasons that users are required to program models is that they often need to implement features (e.g. agent rules) that have never been used before. Programming languages are highly expressive and offer the simplest way to provide the greatest functionality to a user. Some tools do attempt to aid new users by providing simpler languages with which to define agent rules and environmental features (e.g. the gro tool uses a language by the same name that has a highly simplified syntax), but they still require a significant investment of time to learn. As the field of synthetic biology matures, it is likely that easier to use interfaces will emerge. However, at present, programming knowledge is essential to get started.

## Challenges and future directions

The accuracy of agent-based simulations relies on both the agents and virtual environment capturing key features and processes necessary for the emergence of a required collective behaviour. These are not always well understood and so close integration with biologists developing cellular models is essential to ensure that key agent behaviours and environmental factors are present. The Synthetic Biology Open Language (SBOL) [68] and SBML [69] are standards to aid in the exchange of genetic design information and unambiguous definition of biochemical models. Having agent-based tools exploit these formats directly would enable existing curated intracellular models to drive agent behaviours. This would help to validate their function when exposed to realistic extracellular factors, and provide clearer links between model parameters of relevance to the cell biology and desired population-level features. Furthermore, the integration of tools designed to efficiently model the reaction networks inside cells (e.g. Smoldyn [70] or NFsim [71]), and the application of whole-cell models [72] to provide detailed behavioural responses would enable accurate simulations. At present, most tools do not provide these features due to the extensive computational demands of simulating large and complex multi-scale models. However, as the availability of cheap high-performance computing grows, and agent-based tools are updated to better exploit these resources, large multi-scale modelling will become viable.

Many real-world applications of synthetic biology require cells to robustly function within complex environments. Faithfully representing key aspects of these environments is essential to ensure that simulations produce accurate results. The use of microfluidics to study single-cell dynamics has seen significant growth in synthetic biology [73]. Such devices impose intricate boundaries on cells that both physically restricts their movement and controls the flow of nutrients sustaining them. Although the role of fluid flows on natural biofilms has been investigated [74], there is a lack of agent-based modelling tools that incorporate the full range of physical processes that might be experienced by a cell, hampering the ability for them to fully describe many systems of this type.

A significant challenge when capturing the complexity of cellular populations is the typical number of individuals involved. Colonies of bacteria will far exceed 100 million cells. At this size, if only the position of each cell is maintained, over 1 GB of raw data would need to be updated for each time point of a simulation. The execution of models at these scales requires the adoption of efficient parallelizable algorithms and high-performance computing architectures. These allow for a simulation to be broken down into many smaller parts and large numbers of processing units used to solve each concurrently. A shift to highly parallel computing architectures has already taken place in molecular dynamics simulations, leading to huge leaps in the speed and scale of problems that can be solved [75]. Some attempts have also been made to use this approach for synthetic biology applications, e.g. CellModeller [63] exploits graphics processing units (GPUs) to accelerate simulations, but these optimizations often come at the cost of limiting the range of possible agent behaviours and the complexity of the virtual environment. While several of the general-purpose modelling frameworks (e.g. FLAME and Repast) do support these types of large-scale simulation, they also lack the biologically relevant built-in features (e.g. cell growth and simulation of genetic networks) that are critical for the efficient development of synthetic biology-related simulations. To further mitigate some of these computational difficulties, attempts have also been made to employ alternative forms of modelling. Hybrid approaches in which an agent-based model is combined with continuous models has been shown to significantly reduce the computational demands of some forms of simulation [76], and dynamic network-based models can be used to simplify the virtual environment, while still ensuring that interactions between cells are fully captured [77–80].

The large number of agent-based modelling tools raises the question: why do so many exist? This is partially due to historic reasons. As various sub-fields of biology have applied agent-based models, they each have developed tools containing the specific features they require. Although this makes it easier for them to tailor models to their specific needs, it also leads to numerous tools all focused on slightly different problems. It is conceivable that a single tool could eventually encapsulate the functionality of all of these. Some efforts in this direction have already begun with Chaste and BSim being built around a ‘plug-n-play’ architecture where simulations are built from a set of available modules. Because users can also define their own modules from scratch, the functionality of the tool can be easily extended in new ways. Intuitively, it would seem that this type of approach will eventually become the standard. However, this flexibility makes it impossible to highly optimize the interactions between modules. This results in less efficient simulations. Therefore there is always likely to be a range of modelling tools available, especially for specific areas that require the highest performance simulations.

In summary, our knowledge of the inner workings of cells has grown significantly over recent years. This has supported the development of genetically engineered cells able to sustainably produce useful chemicals [4] and implement novel behaviours [1–3,27,28,36,81–83]. Nevertheless, synthetic biology has struggled to effectively scale systems beyond individual cells to the rational engineering of multicellular collective functions. Agent-based modelling offers a way to explore the links between single-cell behaviours and population-level phenomena [9]. This will help to support the next wave of synthetic biology applications that exploit large populations of cells to implement robust functionalities at scale.

## Summary

- Agent-based modelling offers a methodology for simulating the emergence of multicellular behaviors and helps us to better understanding the underlying cellular rules that facilitate these.
- Synthetic biological systems that rely on communication between cells or physical interactions are highly amenable to agent-based modelling and there is growing use of the technique within the field.
- Numerous computational tools exist to support the development of agent-based models for synthetic biology. However, trade-offs in the ease of use and available features mean that careful selection of an appropriate tool is essential.
- Broader use of agent-based modelling will support the scale-up of synthetic biology, allowing not only the creation of new large-scale functions, but also providing insight into how natural systems achieve similar capabilities.

## Abbreviations

DDE: delay differential equation
FLAME: Flexible Large-scale Agent Modelling Environment
GPU: graphics processing unit
ODE: ordinary differential equation
SBML: Systems Biology Markup Language
SBOL: Synthetic Biology Open Language

## References

[1] Roquet N., Soleimany A.P., Ferris A.C., Aaronson S., Lu T.K. (2016). Synthetic recombinase-based state machines in living cells. Science.

[2] Moon T.S., Lou C., Tamsir A., Stanton B.C., Voigt C.A. (2012). Genetic programs constructed from layered logic gates in single cells. Nature.

[3] Danino T., Prindle A., Kwong G.A., Skalak M., Li H., Allen K. (2015). Programmable probiotics for detection of cancer in urine. Sci. Transl. Med..

[4] Paddon C.J., Keasling J.D. (2014). Semi-synthetic artemisinin: a model for the use of synthetic biology in pharmaceutical development. Nat. Rev. Microbiol..

[5] Endy D., Brent R. (2001). Modelling cellular behaviour. Nature.

[6] Kong K.F., Vuong C., Otto M. (2006). *Staphylococcus* quorum sensing in biofilm formation and infection. Int. J. Med. Microbiol..

[7] Waters C.M., Bassler B.L. (2005). Quorum sensing: cell-to-cell communication in bacteria. Annu. Rev. Cell Dev. Biol..

[8] Lai S., Tremblay J., Deziel E. (2009). Swarming motility: a multicellular behaviour conferring antimicrobial resistance. Environ. Microbiol..

[9] Hellweger F.L., Clegg R.J., Clark J.R., Plugge C.M., Kreft J.U. (2016). Advancing microbial sciences by individual-based modelling. Nat. Rev. Microbiol..

[10] Ozbudak E.M., Thattai M., Lim H.N., Shraiman B.I., van Oudenaarden A. (2004). Multistability in the lactose utilization network of *Escherichia coli*. Nature.

[11] Richardson T.O., Gorochowski T.E. (2015). Beyond contact-based transmission networks: the role of spatial coincidence. J. R. Soc. Interface.

[12] Hauert S., Bhatia S.N. (2014). Mechanisms of cooperation in cancer nanomedicine: towards systems nanotechnology. Trends Biotechnol..

[13] McMullin B. (2000). John von Neumann and the evolutionary growth of complexity: looking backward, looking forward. Artificial Life.

[14] Holland J.H., Miller J.H. (1991). Artificial adaptive agents in economic theory. Am. Economic Rev..

[15] Lukas G. (1972). Uses of the LOGO programming language in undergraduate instruction. Proceedings of the ACM annual conference - Volume 2.

[16] Resnick M. (1996). StarLogo: an environment for decentralized modeling and decentralized thinking. Conference Companion on Human Factors in Computing Systems.

[17] Tisue S., Wilensky U. (2004). NetLogo: design and implementation of a multi-agent modeling environment. Proceedings of the Agent 2004 Conference on Social Dynamics: Interaction, Reflexivity and Emergence.

[18] Tesfatsion L., Judd K.L. (2006). Handbook of Computational Economics: Agent-Based Computational Economics.

[19] Axelrod R.M. (1997). The Complexity of Cooperation: Agent-based Models of Competition and Collaboration.

[20] Grimm V., Revilla E., Berger U., Jeltsch F., Mooij W.M., Railsback S.F. (2005). Pattern-oriented modeling of agent-based complex systems: lessons from ecology. Science.

[21] Auchincloss A.H., Diez Roux A.V. (2008). A new tool for epidemiology: the usefulness of dynamic-agent models in understanding place effects on health. Am. J. Epidemiol..

[22] Miller M.B., Bassler B.L. (2001). Quorum sensing in bacteria. Annu. Rev. Microbiol..

[23] Glass L. (2001). Synchronization and rhythmic processes in physiology. Nature.

[24] Kruse K., Jülicher F. (2005). Oscillations in cell biology. Curr. Opin. Cell Biol..

[25] Purcell O., Savery N.J., Grierson C.S., di Bernardo M. (2010). A comparative analysis of synthetic genetic oscillators. J. R. Soc. Interface.

[26] Leibler S., Elowitz M.B. (2000). A synthetic oscillatory network of transcriptional regulators. Nature.

[27] Danino T., Mondragon-Palomino O., Tsimring L., Hasty J. (2010). A synchronized quorum of genetic clocks. Nature.

[28] Din M.O., Danino T., Prindle A., Skalak M., Selimkhanov J., Allen K. (2016). Synchronized cycles of bacterial lysis for *in vivo* delivery. Nature.

[29] Mina P., di Bernardo M., Savery N.J., Tsaneva-Atanasova K. (2013). Modelling emergence of oscillations in communicating bacteria: a structured approach from one to many cells. J. R. Soc. Interface.

[30] Ceroni F., Algar R., Stan G.B., Ellis T. (2015). Quantifying cellular capacity identifies gene expression designs with reduced burden. Nat. Methods.

[31] Gorochowski T.E., van den Berg E., Kerkman R., Roubos J.A., Bovenberg R.A. (2014). Using synthetic biological parts and microbioreactors to explore the protein expression characteristics of *Escherichia coli*. ACS Synth. Biol..

[32] Fernandez-Rodriguez J., Yang L., Gorochowski T.E., Gordon D.B., Voigt C.A. (2015). Memory and combinatorial logic based on DNA inversions: dynamics and evolutionary stability. ACS Synth. Biol..

[33] Gyorgy A., Jiménez J.I., Yazbek J., Huang H.H., Chung H., Weiss R. (2015). Isocost lines describe the cellular economy of genetic circuits. Biophys. J..

[34] Gorochowski T.E., Avcilar-Kucukgoze I., Bovenberg R.A.L., Roubos J.A., Ignatova Z. (2016). A minimal model of ribosome allocation dynamics captures trade-offs in expression between endogenous and synthetic genes. ACS Synth. Biol..

[35] Chen Y., Kim J.K., Hirning A.J., Josić K., Bennett M.R. (2015). Emergent genetic oscillations in a synthetic microbial consortium. Science.

[36] Tamsir A., Tabor J.J., Voigt C.A. (2011). Robust multicellular computing using genetically encoded NOR gates and chemical 'wires'. Nature.

[37] Urrios A., Macia J., Manzoni R., Conde N., Bonforti A., de Nadal E. (2016). A synthetic multicellular memory device. ACS Synth. Biol..

[38] Silva-Rocha R., de Lorenzo V. (2014). Engineering multicellular logic in bacteria with metabolic wires. ACS Synth. Biol..

[39] Gorochowski T.E., Matyjaszkiewicz A., Todd T., Oak N., Kowalska K., Reid S. (2012). BSim: an agent-based tool for modeling bacterial populations in systems and synthetic biology. PLoS One.

[40] Goñi-Moreno A., Amos M., de la Cruz F. (2013). Multicellular computing using conjugation for wiring. PLoS One.

[41] Pascalie J., Potier M., Kowaliw T., Giavitto J.L., Michel O., Spicher A. (2016). Developmental design of synthetic bacterial architectures by morphogenetic engineering. ACS Synth. Biol..

[42] Rudge T.J., Federici F., Steiner P.J., Kan A., Haseloff J. (2013). Cell polarity-driven instability generates self-organized, fractal patterning of cell layers. ACS Synth. Biol..

[43] Cho H., Jönsson H., Campbell K., Melke P., Williams J.W., Jedynak B. (2007). Self-organization in high-density bacterial colonies: efficient crowd control. PLoS Biol.

[44] Lapin A., Klann M., Reuss M., Wittmann C., Krull R. (2010). Multi-scale spatio-temporal modeling: lifelines of microorganisms in bioreactors and tracking molecules in cells. Biosystems Engineering II: Linking Cellular Networks and Bioprocesses.

[45] Husselmann A.V., Hawick K.A. (2013). Simulating growth kinetics in a data-parallel 3D lattice photobioreactor. Model. Simul. Eng..

[46] Hawick K.A., Husselmann A.V.

[47] Sklar E. (2007). Software review: NetLogo, a multi-agent simulation environment. Artificial Life.

[48] Wilensky U.

[49] North M.J., Collier N.T., Ozik J., Tatara E.R., Macal C.M., Bragen M., Sydelko P. (2013). Complex adaptive systems modeling with Repast Simphony. Complex Adapt. Syst. Model..

[50] Collier N., North M. (2013). Parallel agent-based simulation with Repast for high performance computing. Simulation.

[51] Holcombe M., Adra S., Bicak M., Chin S., Coakley S., Graham A.I. (2012). Modelling complex biological systems using an agent-based approach. Integr. Biol..

[52] Berg H.C., Brown D.A. (1972). Chemotaxis in *Escherichia coli* analysed by three-dimensional tracking. Nature.

[53] Emonet T., Macal C.M., North M.J., Wickersham C.E., Cluzel P. (2005). AgentCell: a digital single-cell assay for bacterial chemotaxis. Bioinformatics.

[54] Vladimirov N., Lovdok L., Lebiedz D., Sourjik V. (2008). Dependence of bacterial chemotaxis on gradient shape and adaptation rate. PLoS Comput. Biol..

[55] Kreft J.-U., Booth G., Wimpenny J.W.T. (1998). BacSim, a simulator for individual-based modelling of bacterial colony growth. Microbiology.

[56] Kreft J.-U., Picioreanu C., Wimpenny J.W.T., van Loosdrecht M.C.M. (2001). Individual-based modelling of biofilms. Microbiology.

[57] Picioreanu C., van Loosdrecht M.C.M., Heijen J.J. (1998). A new combined differenital-discrete cellular automaton approach of biofilm modeling: application for growth in gel beads. Biotechnol. Bioeng..

[58] Lardon L.A., Merkey B.V., Martins S., Dötsch A., Picioreanu C., Kreft J.U. (2011). iDynoMiCS: next-generation individual-based modelling of biofilms. Environ. Microbiol..

[59] van Loosdrecht M.C.M., Heijen J.J., Kreft J.-U., Picioreanu C. (2001). Mathematical modelling of biofilm structures. Antonie van Leeuwenhoek.

[60] Picioreanu C., Kreft J.U., van Loosdrecht M.C.M. (2004). Particle-based multidimensional multispecies biofilm model. Appl. Environ. Microbiol..

[61] Fozard J.A., Lees M., King J.R., Logan B.S. (2012). Inhibition of quorum sensing in a computational biofilm simulation. Biosystems.

[62] Wei G., Bogdan P., Marculescu R. (2013). Efficient modeling and simulation of bacteria-based nanonetworks with BNSim. IEEE J. Sel. Areas Commun..

[63] Rudge T.J., Steiner P.J., Phillips A., Haseloff J. (2012). Computational modeling of synthetic microbial biofilms. ACS Synth. Biol..

[64] Jang S.S., Oishi K.T., Egbert R.G., Klavins E. (2012). Specification and simulation of synthetic multicelled behaviors. ACS Synth. Biol..

[65] Mirams G.R., Arthurs C.J., Bernabeu M.O., Bordas R., Cooper J., Corrias A. (2013). Chaste: an open source C++ library for computational physiology and biology. PLoS Comput. Biol..

[66] Pitt-Francis J., Pathmanathana P., Bernabeua M.O., Bordasa R., Coopera J., Fletcherb A.G. (2009). Chaste: a test-driven approach to software development for biological modelling. Comput. Phys. Commun..

[67] Swat M.H., Thomas G.L., Belmonte J.M., Shirinifard A., Hmeljak D., Glazier J.A. (2012). Multi-scale modeling of tissues using CompuCell3D. Methods Cell Biol.

[68] Galdzicki M., Clancy K.P., Oberortner E., Pocock M., Quinn J.Y., Rodriguez C.A. (2014). The Synthetic Biology Open Language (SBOL) provides a community standard for communicating designs in synthetic biology. Nat. Biotechnol..

[69] Hucka M., Finney A., Sauro H.M., Bolouri H., Doyle J.C., Kitano H. (2003). The systems biology markup language (SBML): a medium for representation and exchange of biochemical network models. Bioinformatics.

[70] Andrews S.S., Addy N.J., Brent R., Arkin A.P. (2010). Detailed simulations of cell biology with Smoldyn 2.1. PLoS Comput. Biol..

[71] Sneddon M.W., Faeder J.R., Emonet T. (2011). Efficient modeling, simulation and coarse-graining of biological complexity with NFsim. Nat. Methods.

[72] Karr J.R., Sanghvi J.C., Macklin D.N., Gutschow M.V., Jacobs J.M., Bolival B. (2012). A whole-cell computational model predicts phenotype from genotype. Cell.

[73] Ferry M.S., Razinkov I.A., Hasty J. (2011). Microfluidics for synthetic biology: from design to execution. Methods Enzymol.

[74] Alpkvist E., Klapper I. (2007). Description of mechanical response including detachment using a novel particle model of biofilm/flow interaction. Water Sci. Technol..

[75] Stone J.E., Hardy D.J., Ufimtsev I.S., Schulten K. (2010). GPU-accelerated molecular modeling coming of age. J. Mol. Graph. Model..

[76] Cilfone N.A., Kirschner D.E., Linderman J.J. (2015). Strategies for efficient numerical implementation of hybrid multi-scale agent-based models to describe biological systems. Cell. Mol. Bioeng..

[77] Gorochowski T.E., Bernardo M.D., Grierson C.S. (2012). Evolving dynamical networks: a formalism for describing complex systems. Complexity.

[78] DeLellis P., Bernardo M., Gorochowski T., Russo G. (2010). Synchronization and control of complex networks via contraction, adaptation and evolution. IEEE Circuits Syst. Mag..

[79] Gross T., Blasius B. (2008). Adaptive coevolutionary networks: a review. J. R. Soc. Interface.

[80] Holme P., Saramäki J. (2012). Temporal networks. Phys. Rep..

[81] Mondragón-Palomino O., Danino T., Selimkhanov J., Tsimring L., Hasty J. (2011). Entrainment of a population of synthetic genetic oscillators. Science.

[82] Farzadfard F., Lu T.K. (2014). Genomically encoded analog memory with precise *in vivo* DNA writing in living cell populations. Science.

[83] Prindle A., Samayoa P., Razinkov I., Danino T., Tsimring L.S., Hasty J. (2012). A sensing array of radically coupled genetic ‘biopixels’. Nature.

[84] Quinn J.Y., Cox 3rd, R.S., Adler A., Beal J., Bhatia S., Cai Y. (2015). SBOL visual: a graphical language for genetic designs. PLoS Biol.

[85] Der B.S., Glassey E., Bartley B.A., Enghuus C., Goodman D.B., Gordon D.B., Voigt C.A., Gorochowski T.E. (2016). DNAplotlib: programmable visualization of genetic designs and associated data. ACS Synth. Biol..

